# Impact of COVID-19 Infection and Persistent Lingering Symptoms on Patient Reported Indicators of Nutritional Risk and Malnutrition

**DOI:** 10.3390/nu14030642

**Published:** 2022-02-02

**Authors:** Rachel R. Deer, Erin Hosein, Madelyn Harvey, Trang Nguyen, Amy Givan, Megan Hamilton, Kayla Turner, Rae Kretzmer, Madeline Rock, Maria C. Swartz, Justin Seashore, Blair Brown, Christopher Messenger

**Affiliations:** 1 Department of Nutrition, Metabolism, and Rehabilitation Sciences, University of Texas Medical Branch, Galveston, TX 77555, USA; rachelrdeer@gmail.com (R.R.D.); trangngu@utmb.edu (T.N.); amgivan@utmb.edu (A.G.); meghamil@utmb.edu (M.H.); kkturner@utmb.edu (K.T.); 2 Sealy Center on Aging, University of Texas Medical Branch, Galveston, TX 77555, USA; meharvey@utmb.edu (M.H.); rmkretzm@utmb.edu (R.K.); 3 Department of Anthropology, Indiana University, Bloomington, IN 47405, USA; erhosein@iu.edu; 4 School of Medicine, University of Texas Medical Branch, Galveston, TX 77555, USA; marock@utmb.edu (M.R.); Justin.b.seashore@kp.org (J.S.); 5 Department of Pediatrics-Research, University of Texas MD Anderson Cancer Center, Houston, TX 77030, USA; mchang1@mdanderson.org

**Keywords:** dietary recall, COVID-19, coronavirus, eating pattern, malnutrition

## Abstract

Persistent malnutrition after COVID-19 infection may worsen outcomes, including delayed recovery and increased risk of rehospitalization. This study aimed to determine dietary intakes and nutrient distribution patterns after acute COVID-19 illness. Findings were also compared to national standards for intake of energy, protein, fruit, and vegetables, as well as protein intake distribution recommendations. Participants (≥18 years old, *n* = 92) were enrolled after baseline visit at the Post-COVID Recovery Clinic. The broad screening battery included nutritional assessment and 24-h dietary recall. Participants were, on average, 53 years old, 63% female, 69% non-Hispanic White, and 59% obese/morbidly obese. Participants at risk for malnutrition (48%) experienced significantly greater symptoms, such as gastric intestinal issues, loss of smell, loss of taste, or shortness of breath; in addition, they consumed significantly fewer calories. Most participants did not meet recommendations for fruit or vegetables. Less than 39% met the 1.2 g/kg/day proposed optimal protein intake for recovery from illness. Protein distribution throughout the day was skewed; only 3% met the recommendation at all meals, while over 30% never met the threshold at any meal. Our findings highlight the need for nutritional education and support for patients to account for lingering symptoms and optimize recovery after COVID-19 infection.

## 1. Introduction

Malnutrition is a serious health problem among all ages, especially older adults. The prevalence of malnutrition is substantially greater in patients admitted to a hospital acute care setting, ranging from 23 to 60% depending on the screening technique utilized and the severity of disease. The more intensive the care, the more probable a patient may become malnourished [1]. Malnutrition is known to have a significant impact on the immune system, resulting in reduced immunological responses and an increased risk of infection and disease severity [2]. In a variety of diseases, body composition, particularly low lean mass, and high adiposity, has repeatedly been associated with a poor prognosis [2].

Similarly, acute COVID-19 illness can lead to nutritional decline for many reasons. Patients with COVID-19 infection often experience symptoms that can lead to decreased nutritional intake such as loss of taste and smell, nausea, and vomiting [3,4]. During acute COVID-19 illness, severe inflammatory response has been observed, which increases nutritional needs, in some cases to more than two times the caloric requirements determined by predictive equations [5]. Depending on the severity of the illness, increased nutritional needs can vary from days, weeks, or years to promote recovery [6]. Consuming enough nutrients to counteract this hypermetabolic state and prevent malnutrition can be challenging for patients. Studies have shown that evenly distributed protein intake may support muscle protein synthesis (MPS) in recovery, which is especially essential in patients at risk of muscle catabolism due to malnutrition when intake is suboptimal and physical activity may be reduced [7,8,9,10]. Additionally, patients with severe illness may need ventilator support, which prevents oral nutrition intake and may necessitate enteral nutrition support. Even with enteral nutrition, patients may fail to meet their nutritional needs due to late enteral initiation, disruption of feeds due to patient procedures, intolerance of enteral feeds, or feeding tube displacement [5]. An appropriate diet is extremely important for the normal function of the immune system and to aid in recovery after an acute illness such as COVID-19 infection. Protein–energy malnutrition, as well as shortages in certain single nutrients, are strongly linked to an increased risk of infectious diseases and delays in recovery from illness [2].

Patients with mild COVID-19 who are treated at home are also at risk of malnutrition [11]. Clinical symptoms experienced by COVID-19 survivors, particularly fatigue, dyspnea, and eating difficulties (nausea, vomiting, changes in smell and taste, and trouble breathing) are common and create difficulties in maintaining adequate nutritional intake [3]. Home confinement and COVID-19 symptoms may limit physical activity, resulting in a loss of lean mass. Even in non-hospitalized patients, these factors, along with a systemic inflammatory response, may result in malnutrition. In addition, persistent lingering gastrointestinal symptoms experienced with long-COVID may negatively impact dietary intake, further delaying recovery from acute illness. However, little data or research currently exist on the influence of COVID-19 on nutritional status [11].

The primary aim of this study was to determine dietary intakes and nutrient distribution patterns after acute COVID-19 illness. We also compared these findings to national standards for the intake of energy, protein, fruit, and vegetables, as well as the Healthy Eating Index (HEI).

## 2. Materials and Methods

### 2.1. Study Cohort

This study was approved by the University of Texas Medical Branch (UTMB) Institutional Review Board. Verbal informed consent was obtained from each participant before any study procedures. Participants were recruited into this study from the Post-COVID Recovery Clinic (PCRC) at the University of Texas Medical Branch. Patients eligible for inclusion met the following general criteria: (1) positive COVID-19 PCR or antibody test; (2) completed baseline appointment with PCRC clinic; (3) aged 18 years or older, and (4) able to consent to participate in the study. The PCRC evaluated all patients via a standard screening tool during 1-h-long telehealth or in-person appointments. The broad screening battery aimed to obtain a broad picture of all symptoms and complex needs. Measures included: acute COVID-19 history, a targeted range-of-symptoms questionnaire, and a validated nutritional assessment (patient-generated subjective global assessment, PG-SGA). Patients were considered at risk for malnutrition if the PG-SGA score was ≥4 [12].

Detailed nutrition analyses were collected and analyzed using a guided 24-h diet recall (Automated Self-Administered, ASA-24). ASA24 utilizes the Food and Nutrient Database for Dietary Studies (FNDDS) and the United States Department of Agriculture’s (USDA) Food Pyramid Equivalents Database. Participants were asked to provide at least 1 day of a diet log collected on “a typical day of eating”. When multiple recalls were collected for a single time point, diet recalls were averaged together to create one entry for each participant for analysis.

#### 2.1.1. Acceptable Macronutrient Distribution Ranges (AMDR)

The AMDR for the study population was determined by calculating kilocalories from grams of protein, fat, and carbohydrate using the accepted 4, 9, and 4 kilocalorie/g benchmarks of these macronutrients, respectively. The kilocalories provided by each macronutrient were divided by the total kilocalories for the day to determine the percent of calories consumed from each macronutrient.

#### 2.1.2. Energy, Vegetable, and Fruit Needs

Estimated needs were based on the 2020 US dietary guidelines [13]. Total mean kilocalories, total mean fruit, and total mean vegetable intake from participants’ dietary recalls were calculated and described in comparison with age and gender standards. 

#### 2.1.3. Protein

Average protein intake (g/kg) was calculated for each participant using protein intake (g) divided by ideal body weight (IBW). IBWs are often used when determining protein needs. IBW was calculated by adjusting each participant’s actual weight (kg) to the nearest weight that would give the participant a body mass index (BMI) between 22 and 27 kg/m^2^, which is the BMI range associated with lower mortality in older adults [14,15]. The average intake was compared to the current recommended dietary allowance (RDA) of 0.8 g/kg for adults and the optimal protein intake of 1.2 g/kg, which has been proposed in the literature to improve health outcomes in older adults with acute or chronic diseases [16,17].

#### 2.1.4. Healthy Eating Index

The HEI-2015 was used to assess participants’ diet quality from ASA-24 diet recalls. The HEI-2015 measures overall diet quality based on consumption of total fruit, greens/beans, whole grains, dairy, protein, seafood/plant protein, fatty acids, refined grains, sodium, and empty calories. Possible HEI-2015 scores range from 0 to 100 points based on the sum of each dietary component score [18]. HEI scores above 80 were scored as having “good” diet quality. HEI of 51–80 indicated “fair” diet quality, and scores of less than 51 were scored as “poor” diet quality. The percentages of participants falling into each of these categories were determined.

The HEI-2015 scoring was calculated using the SAS program created by the National Cancer Institute. The population ratio method was used to calculate estimated population means of HEI-2015 scores, which is recommended to compare component scores with recommended standards. If a respondent had more than one ASA-24 input for a single day, the intake data was averaged. More details regarding the method and calculations of the HEI-2015 population ratio method and calculations can be found at https://epi.grants.cancer.gov/hei/hei-methods-and-calculations.html (accessed on 6 December 2021).

A radar plot was used to visualize the HEI-2015 component scoring, where 100 or the outer wheel represents a perfect component score. The maximum score is presented in percentage to show the percent of the maximum score achieved from the HEI-2015 standard component scoring and to make the component scores comparable across food components (e.g., [a score of 4.19 for total vegetables/5 maximum achievable score for total fruits] × 100 = 83.8%).

### 2.2. Meal Patterns

Energy and protein intakes were assessed at each mealtime. Mealtimes were assigned as follows: breakfast (4:00–9:59), lunch (10:00–15:59), and dinner (16:00–21:59). To prevent potentially skewing meals, meals were divided into equal six-hour windows, creating an additional category for the remaining overnight timeframe (22:00–3:59). Differences in energy (kcal) and protein (g) between meals were compared. Meal protein consumption was also compared to the optimal 0.4 g protein/kg/meal threshold suggested for adults and older adults that are at risk of catabolic stress or malnutrition during a period of energy deficiency [7,9,19].

### 2.3. Statistical Analyses

Continuous variables were compared across malnutrition risk (PG-SGA Score <4 vs. ≥4) using a two-sided *t*-test. A Chi-square test and a Kruskal–Wallis Chi-square test were conducted to compare categorical variables across malnutrition risk for normally distributed variables and non-normally distributed variables. A one-sample *t*-test was conducted to compare the average protein consumption to body weight ratio to the current RDA protein consumption recommendations and proposed protein consumption recommendations. A one-sample *t*-test was also conducted to compare the average protein meal consumption to ideal body weight ratio to the proposed 0.4 g/kg threshold. A two-sample *t*-test was conducted to compare kcal intake between participants who experienced symptoms and those who experienced no symptoms. Normality assumptions and outliers for covariates were assessed using Shapiro–Wilk tests and boxplots. All statistical analyses were performed using SAS (version 9.4; SAS Institute Inc., Cary, NC, USA) or R (version 4.1.1;R Core Team, Vienna, Austria). The radar plot was created using Microsoft Excel and PowerPoint (version 2019; Microsoft, Redmond, WA, USA) for Windows. A *p*-value of less than 0.05 was considered statistically significant.

## 3. Results

A total of 130 dietary logs were collected from 92 participants with 41.3% completing two dietary records. An average of both records was used for all analyses when participants had two completed logs. Forty-four participants (47.8%) were categorized as at risk of malnutrition (PG-SGA ≥ 4).

Participant demographics are shown in Table 1. The mean age was 53.7 ± 13.7 years, 65.2% were female, and 69.2% were non-Hispanic White. According to body mass index (BMI) classification, 31.5% were morbidly obese, 27.2% were obese, 28.3% were overweight, 10.9% were normal weight, and 2.2% were underweight. The time from COVID-19 positive date to clinic visit was 96.8 ± 86.4 days. There were no significant differences across participant demographic characteristics by risk of malnutrition.

Table 2 shows the number of symptom complaints at the clinic visits. Participants had, on average, 6.6 ± 3.9 lingering symptoms (range 0–12). The most common symptoms were fatigue (81.5%), dyspnea on exertion (79.4%), and weakness (67.4%). Participants at risk of malnutrition (PG-SGA ≥ 4) experienced a greater number of the following symptoms: diarrhea (20.5% vs. 6.5%, *p* = 0.043), abdominal pain (27.3% vs. 6.3%, *p* = 0.006), loss of smell (31.8% vs. 10.4%, *p* = 0.011), loss of taste (34.1% vs. 12.5%, *p* = 0.014), congestion (43.2% vs. 22.9% *p* = 0.038), and dyspnea (88.6% vs. 77.1%, *p* = 0.035).

Participants who reported experiencing gastrointestinal symptoms (i.e., loss of smell, loss of taste, nausea, vomiting, diarrhea, and abdominal pain) had significantly different average energy consumption compared to participants who reported experiencing no symptoms (1530 ± 729 kcal vs. 1744 ± 710 kcal, *p* = 0.012).

### 3.1. Group Nutrient Intakes vs. Recommendations

#### 3.1.1. AMDR Average Macronutrient Distribution Range

On average, the study participants’ macronutrient-distribution range was 17.4% protein, 37.4% fat, and 45.4% carbohydrates. Protein and carbohydrates were within the recommended AMDR range [13]; however, fat exceeded the recommendation (20–35%).

#### 3.1.2. Energy, Vegetable, and Fruit

Table 3 reports the current age and sex specific recommendations for energy, fruit, and vegetable and the average intake of study participants. Females ≥30 years of age (*n* = 55) had, on average, lower total fruit consumed per day (cup eq/d) than recommended (0.7 ± 0.9 vs. 1.5 cup eq/d, *p* < 0.0001) and lower total vegetables consumed per day (cup eq/d) than recommended (1.5 ± 1.3 vs. 2 cup eq/d, *p* = 0.004). Females between the ages of 19 and 30 years (*n* = 5) had lower total vegetables consumed per day than recommendations (1.1 ± 0.6 vs. 2.5 cup eq/day, *p* = 0.008). Males ≥ 60 years of age (*n* = 14) had, on average, lower total fruit consumed per day (cup eq/d) than recommended (0.7 ± 0.6 vs. 2 cup eq/d, *p* < 0.0001) and lower total vegetables consumed per day (cup eq/d) than recommended (1.7 ± 1.2 vs. 2.5 cup eq/d, *p* = 0.034). Males between the ages of 31 and 59 (*n* = 16) had lower total fruit consumed per day (cup eq/d) than recommended (0.8 ± 1.1 vs. 2 cup eq/d, *p* = 0.0005) and lower total vegetables consumed per day (cup eq/d) than recommended (1.4 ± 0.8 vs. 3 cup eq/d, *p* < 0.0001).

#### 3.1.3. Protein

Table 4 reports the current recommendation for protein compared to the current US dietary guidelines (0.8 g/kg) [13] and the proposed standard (1.2 g/kg) [17] for older adults. To account for the high percentage of obese patients in the sample, IBW was used for all protein analyses presented (see Supplementary Materials for non-IBW comparisons; Figure S1: Protein consumption by meal time (g protein/kg BW). Table S1: Protein consumption meeting per meal recommendation (g/kg BW) by meal time. Table S2: Average protein consumption to weight comparison of current recommendations (g/kg)). The average protein consumption to body weight (0.8 ± 0.4g/kg) was significantly lower than the proposed standard for protein consumption to bodyweight of 1.2 g/kg (*p* < 0.0001). The average protein consumption to ideal body weight (0.98 ± 0.5) was significantly higher than the current RDA standard of 0.8 g/kg protein consumption (*p* = 0.0003). The average protein consumption to ideal body weight was significantly lower than the proposed standards of 1.2 g/kg for protein consumption to ideal body weight (*p* < 0.0001).

#### 3.1.4. Healthy Eating Index

**Figure 1:**
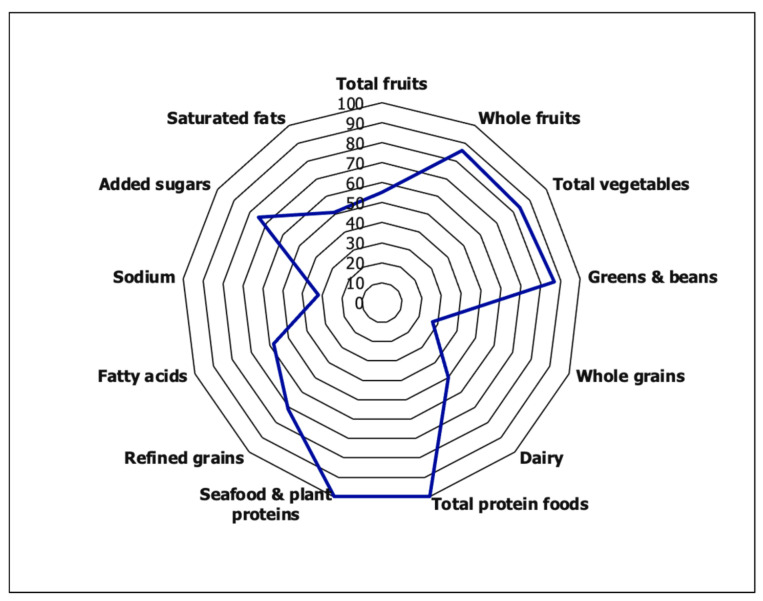
Healthy Eating Index mean component scores.

HEI scores ranged from 29 to 89 points. Of the study population, 4% fell into the category of “good” diet quality, 51% “fair”, and 45% “poor”. The estimated population (61.9 ± 2.3) was lower than the maximum achievable score of 100. The results of the Radar plot are shown in Figure 1. The mean component scores for total protein foods and seafood and plant proteins had a perfect HEI-2015 score (100%). The second highest HEI-2015 scores were greens and beans (87%), whole fruits (86%), and total vegetables (84%), followed by added sugars (75%), refined grains (71%), fatty acids (58%), total fruits (55%), saturated fats (51%), and dairy (50%). The lowest mean component HEI-2015 scores were sodium (32%) and whole grains (27%).

### 3.2. Meal Patterns

#### 3.2.1. Daily Energy Distribution

Participants consumed an average of 1707.8 ± 720.3 kcal per day with a range from 178.9 to 4443.3 daily kcal. This was distributed over meals as follows: breakfast 321.1 ± 272.6 kcal, lunch 521.1 ± 374.9 kcal, dinner 815.7 ± 507.6 kcal, and other 49.9 ± 174.6 kcal.

#### 3.2.2. Daily Protein Distribution

**Figure 2:**
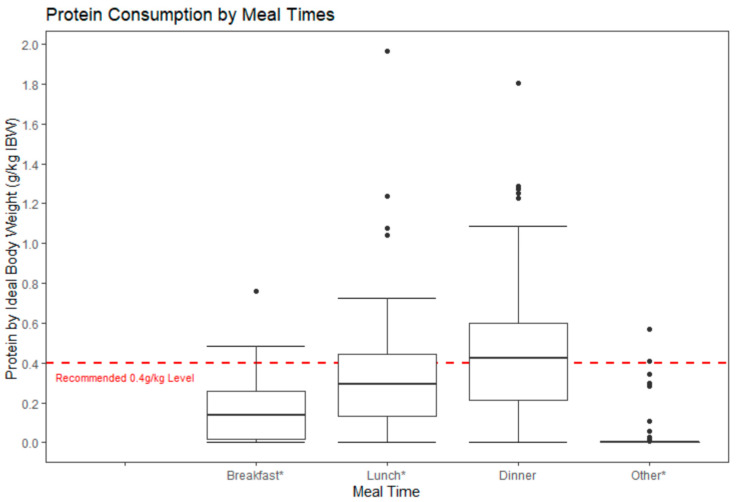
Protein consumption by meal time (g/kg IBW). * indicates statistical significance (*p*-value < 0.05).

Protein consumption per kilogram IBW (g/kg IBW) was significantly different at breakfast (0.2 ± 0.2, *p* < 0.0001), lunch (0.3 ± 0.3, *p* = 0.03), and other (0.02 ± 0.1, *p* < 0.001) than the 0.4 g/kg threshold (Figure 2). On average, protein consumption at dinner exceeded the 0.4 g/kg threshold but was not statistically different (0.5 ± 0.3, *p* > 0.05). The presence of outliers existed in each meal category, potentially positively skewing the average protein consumption (g/kg IBW); however, these averages were still below the recommended 0.4 g/kg threshold at breakfast, lunch, and other. Table 5 shows only 31.5% and 8.7% of the participants met the 0.4 g/kg IBW threshold at lunch and breakfast, while most participants met the threshold at dinner (52.2%). Of the 92 participants, 3 participants (3.3%) met the 0.4 g/kg IBW threshold for three meals, 20 (21.7%) met the threshold for two meals, 64 (69.6%) met it for at least one meal, and 28 (30.4%) of the participants did not meet the threshold at any meal.

## 4. Discussion

To our knowledge, the current study is the first to investigate trends in dietary intake after acute COVID-19 infection. The main findings include: (a) a high percentage of patients after COVID-19 were at risk for malnutrition; (b) participants at risk for malnutrition reported having significantly higher numbers of symptoms of diarrhea, abdominal pain, loss of smell, loss of taste, congestion, or shortness of breath; (c) participants with gastrointestinal symptoms consumed significantly less calories; (d) the majority of participants had, on average, low consumption of fruit or vegetables; (e) while average protein consumption was significantly higher than the current RDA of 0.8 g/kg, less than 40% of participants met the recommendation of 1.2 g/kg; (f) the distribution of protein was skewed throughout the meals and over 30% of participants never met the per-meal protein recommendation of 0.4 g of protein/kg/meal to optimally stimulate muscle protein synthesis.

Participants in our study were, on average, over 3 months post-positive-COVID-19 infection and fell into the long-COVID category. However, most research to date looking at malnutrition or malnutrition risk post-COVID-19 infection has had a short follow-up time of one month or less [11,21,22]. A study by Bedock et al. followed 91 patients hospitalized for COVID-19 infection during admission and 30 days post-discharge and found that 28.6% of patients were malnourished, based on the Global Leadership Initiative on Malnutrition (GLIM) criteria, 30 days after discharge [21]. Similarly, another study of 288 patients hospitalized for COVID-19 found that 33.3% of patients were malnourished based on GLIM criteria at 30 days after discharge [22]. In contrast, Filippo et al. used the Mini Nutritional Assessment (MNA) to assess for malnutrition and found that 54.7% of patients were at risk for malnutrition, but only 6.6% were malnourished when evaluated in an outpatient clinic at a median of 23 days post-discharge for COVID-19 infection [11]. A study by Gerard et al. followed 288 COVID-19 patients discharged from the hospital at 30 days post-discharge and six months post-discharge to evaluate the presence of malnutrition. At 30 days, 47.2% of patients evaluated were malnourished, and at 6 months, 36% of the patients previously identified with malnutrition still had malnutrition based on GLIM criteria [23].

The prevalence of malnutrition in our population was quite high, at 48%, which is not surprising since many of the acute and lingering symptoms of COVID-19 affect the gastrointestinal system [4]. We found that participants at risk for malnutrition experienced significantly greater symptoms of diarrhea, abdominal pain, loss of smell, loss of taste, congestion, or shortness of breath, and those who experienced lingering gastrointestinal symptoms consumed significantly fewer calories on average. This is concerning for both acute and more long-term COVID-19 outcomes. Recent research has shown that malnutrition prolongs hospitalization in COVID-19 patients by almost 12 days [24,25]. In addition, nutritional risk is associated with higher risk of developing severe COVID-19 infection [26].

Very little research exploring the eating patterns of patients with long-COVID has been published to date. Most of the research on long-COVID eating patterns that is currently available has focused on user-generated text posted by long-COVID patients on the internet looking for support and advice from other COVID-19 survivors [27,28]. Patients with long-COVID complain of diverse impacts of lingering symptoms on food preferences, appetite and intake, and weight. Many patients report that altered taste or smell has changed their food preferences, making previously enjoyed foods unappealing and vice versa. People with long-COVID often complain of decreased appetite, which may lead to less food intake and weight loss. Conversely, some state that since COVID-19 infection, they feel insatiable or need to consume more food to “hit the spot” for cravings, which can lead to eating more and gaining weight [27]. Patients also have reported trying different diets such as elimination diets, ketogenic diets, and intermittent fasting in an attempt to find dietary strategies to help improve long-COVID symptoms [28]. It is important to note that while some common COVID-19 symptoms, such as nausea or fatigue, may lead to a loss of appetite and reduced food intake, the reverse causal relationship, in which a decline in food intake leads to nausea and fatigue, cannot be ruled out. Our research expands upon the current knowledge of long-COVID eating patterns by providing a more objective and systematic analysis of what patients are consuming post-COVID.

We closely examined protein consumption as it has been shown to play a critical role in recovery from acute illness. The current RDA for protein, 0.8 g/kg/day, has been argued to not be optimal to optimize recovery. The International PROT-AGE Study Group recommends 1–1.2 g/kg/d for healthy older adults to help maintain and regain lean body mass and function, and up to 1.2–1.5 g/kg/d for adults with acute or chronic diseases [17]. Our participants averaged 0.98 g protein/kg IBW per day. While this amount was significantly higher than the RDA of 0.8 g/kg/d, when compared to the higher 1.2 g/kg/d recommendation, only 39% met the recommendation. Consistent with previous reports [7,14], we found that our participants distributed their daily protein unevenly across the designated mealtimes, with the least amount of protein at breakfast and the most at dinner. Moreover, 30% of participants did not meet the 0.4 g protein/kg/meal threshold at any meal and only 3% consumed enough protein to meet that threshold at every meal. More research is needed to see if this trend is common in patients with long-COVID, though adding moderate amounts of protein to low protein meals, traditionally breakfast and lunch, may be one intervention that could assist in recovery by simulating MPS and fighting catabolism of muscle, which may be common in prolonged suboptimal energy intake and decreased physical activity [7,8,9,10].

This study also has some limitations. The relatively small sample did not allow us to stratify the dietary intakes relative to comorbidities and activity level. Further, the small sample size limited the ability to calculate subgroup population ratios to compare age- and sex- specific recommendations for fruit and vegetable consumption, thus reducing the power of the difference between average intake of energy, fruit, and vegetables with standard recommendations. For example, there were two male participants between the ages of 19 and 30. Our results highlight the high risk of malnutrition in patients long after recovering from acute COVID-19 infection, and the need for future larger and more detailed nutritional studies in this patient population. A second weakness was the collection of a single-day or two-day dietary records. While we asked participants to complete their dietary records on a “typical day of eating”, with a small dataset, we may have not collected usual eating patterns in some participants. However, to minimize the effects of measurement error in collecting dietary records, we calculated estimated population means for the Healthy Eating Index for a single day using standardized code that can be compared across studies. We standardized the data collection and analyses using the ASA24 software. Another weakness was that without “pre-COVID” baseline dietary intake, we were unable to assess if these poor dietary habits were pre-existing or if acute COVID-19 infection led to a change in dietary intake patterns.

## 5. Conclusions

To our knowledge, this is the first study to analyze trends in dietary intake after acute COVID-19 infection. The finding that the participants did not meet the current nutrient intake recommendations is concerning. Nutrition tends to be overlooked during and after hospitalization, likely due to more pressing health problems present during hospital admission. However, the importance of adequate nutrition for the optimization of recovery cannot be overstated. Our findings highlight the need for better nutritional education and support for patients to account for lingering symptoms and optimize recovery from acute COVID-19 infection.

## Figures and Tables

**Table 1:** Demographic characteristics of the final sample by malnutrition risk ^α^ (*n* = 92).

Variable	Total	Not at-Risk for Malnutrition (PG-SGA < 4)	At-Risk for Malnutrition (PG-SGA ≥ 4)	*p*-Value
N (%)	92	48 (52.2%)	44 (47.8%)	
Age (years), mean ± SD	53.7 ± 13.7	54.9 ± 11.9	52.5 ± 15.5	0.400
Female	60 (65.2%)	29 (60.4%)	31 (70.5%)	0.313
Race/Ethnicity				0.377
White, non-Hispanic	63 (69.2%)	37 (77.1%)	26 (60.5%)	
White, Hispanic	16 (17.6%)	6 (12.5%)	10 (23.3%)	
Black, non-Hispanic	9 (9.9%)	4 (8.3%)	5 (11.6%)	
Other	3 (3.3%)	1 (2.1%)	2 (4.7%)	
BMI *				0.369
Underweight	2 (2.2%)	0	2 (2.2%)	
Healthy	10 (10.9%)	6 (12.5%)	4 (9.1%)	
Overweight	26 (28.3%)	13 (27.1%)	13 (29.6%)	
Obese	26 (27.2%)	11 (22.9%)	14 (31.8%)	
Morbidly Obese	29 (31.5%)	18 (37.5%)	11 (25.0%)	

Notes: *p*-value < 0.05 indicates statistical significance. SD, standard deviation; BMI, body mass index (kg/m^2^); PG-SGA, patient-generated subjective global assessment. Race/Ethnicity other (Asian, Hispanic); Underweight = BMI < 20 kg/m^2^, Healthy = BMI 20–24.9 kg/m^2^, Overweight = BMI 25–29.9 kg/m^2^, Obese BMI 30–34.9 kg/m^2^, Morbidly obese = BMI > 35 kg/m^2^. * Kruskal–Wallis Chi-square test conducted. ^α^ Based on FNIH cut points [20].

**Table 2:** Symptoms of final sample by malnutrition risk (*n* = 92).

Symptom	Total *N* = 92	Not at-Risk for Malnutrition (PG-SGA < 4; *n* = 48)	At-Risk for Malnutrition (PG-SGA ≥ 4; *n* = 44)	*p*-Value
Vomiting	6 (6.5%)	2 (4.2%)	4 (9.1%)	0.336
Diarrhea	12 (13.0%)	3 (6.3%)	9 (20.5%)	0.043
Sore Throat	15 (16.3%)	5 (10.4%)	10 (22.7%)	0.110
Abdominal pain	15 (13.0%)	3 (6.3%)	12 (27.3%)	0.006
Loss of smell	19 (20.7%)	5 (10.4%)	14 (31.8%)	0.011
Nausea	20 (21.7%)	7 (14.6%)	13 (29.6%)	0.082
Loss of taste	21 (22.8%)	6 (12.5%)	15 (34.1%)	0.014
Congestion	30 (32.6%)	11 (22.9%)	19 (43.2%)	0.038
Shortness of breath at rest	35 (38.0%)	19 (39.6%)	16 (36.4%)	0.751
Weakness	62 (67.4%)	31 (64.6%)	31 (70.5%)	0.549
Dyspnea	73 (79.4%)	34 (70.8%)	39 (88.6%)	0.035
Fatigue	75 (81.5%)	37 (77.1%)	38 (86.4%)	0.216

Notes: *p*-value < 0.05 indicates statistical significance across groups. PG-SGA, patient-generated subjective global assessment.

**Table 3:** Average intake of energy, fruit, and vegetables.

**Energy (kcal/day) Recommendation**		**Average Consumption**	**Range**	**Met Recommendation**
Females 31+ (*n* = 55)	1600	1456 ± 574	(179–2874)	36%
Females 19–30 (*n* = 5)	1800	2006 ± 420	(1447–2584)	80%
Males 60+ (*n* = 14)	2000	2247 ± 814	(633–4443)	64%
Males 31–59 (*n* = 16)	2200	1917 ± 812	(672–3070)	31%
Males 19–30 (*n* = 2)	2400	2430 ± 699	(1935–2924)	50%
**Fruit (cup eq/day) Recommendation**		**Average Consumption**	**Range**	**Met Recommendation**
Females 31+ (*n* = 55)	1 ½	0.69 ± 0.91	(0–3.48)	20%
Females 19–30 (*n* = 5)	1 ½	0.89 ± 0.66	(0–1.52)	20%
Males 60+ (*n* = 14)	2	0.68 ± 0.64	(0–1.99)	0%
Males 31–59 (*n* = 16)	2	0.80 ± 1.09	(0–3.95)	19%
Males 19–30 (*n* = 2)	2	2.58 ± 2.79	(0.61–4.55)	50%
**Vegetable (cup eq/day) Recommendation**		**Average Consumption**	**Range**	**Met Recommendation**
Females 31+ (*n* = 55)	2	1.47 ± 1.31	(0–6.39)	24%
Females 19–30 (*n* = 5)	2 ½	1.10 ± 0.64	(0.40–2.02)	0%
Males 60+ (*n* = 14)	2 ½	1.74 ± 1.20	(0.14–4.78)	14%
Males 31–59 (*n* = 16)	3	1.37 ± 0.82	(0–3.26)	6%
Males 19–30 (*n* = 2)	3	1.86 ± 1.15	(1.04–2.68)	0%

Notes: Average consumption is the total meal consumption. kcal, kilocalorie; eq, equivalent.

**Table 4:** Average protein consumption to weight comparison of current recommendations (*n* = 92).

Recommendations (Protein g/kg IBW)	Average Consumption	Range	Met Recommendations, *n* (%)	*p*-Value
Current RDA (0.8 g/kg IBW) [13]	0.98 ± 0.5	0.45–3.02	59 (64.1%)	0.0003
Proposed standard (1.2 g/kg IBW) [17]	0.98 ± 0.5	0.45–3.02	23 (39%)	<0.0001

Notes: *p*-value < 0.05 indicates statistical significance across groups. RDA, recommended daily allowance; IBW, ideal body weight; kg, kilograms.

**Table 5:** Protein consumption by meal time (*n* = 92).

Meal	Average Kcal Consumption ± SD	Range Kcal	Average Protein Consumption (g) ± SD	Range Protein (g)	Met Per Meal Recommendation ≥ 0.4g/kg IBW; *n* (%)
Breakfast	321.1 ± 272.6	0–1563.8	12.1 ± 11.6	0–59.2	8 (8.7%)
Lunch	521.1 ± 374.9	0–2026.9	24.4 ± 23.6	0–145.9	29 (31.5%)
Dinner	815.7 ± 507.6	0–2933.7	34.8 ± 25.5	0–140.9	48 (52.2%)
Other	49.9 ± 174.6	0–993.5	1.8 ± 7.4	0–51.4	2 (2.2%)

Notes: SD, standard deviation; g, grams; kcal, kilocalorie; g/kg IBW, grams per kilograms Ideal Body Weight.

## Data Availability

The data presented in this study are available on request. The data are not publicly available due to privacy protection reasons.

## Supplementary Materials

The following are available online at https://www.mdpi.com/article/10.3390/nu14030642/s1, Figure S1: Protein consumption by meal time (g protein/kg BW). Table S1: Protein consumption meeting per meal recommendation (g/kg BW) by meal time. Table S2: Average protein consumption to weight comparison of current recommendations (g/kg).
