# Supplementary material for: Impact of COVID-19 Infection and Persistent Lingering Symptoms on Patient Reported Indicators of Nutritional Risk and Malnutrition
Source: Nutrients. 2022 Feb 2;14(3):642. doi: 10.3390/nu14030642 (PMC8839070; doi:10.3390/nu14030642)
Supplement: Supplementary file 1 [file nutrients-14-00642-s001.zip › nutrients-1560991-supplementary.pdf]

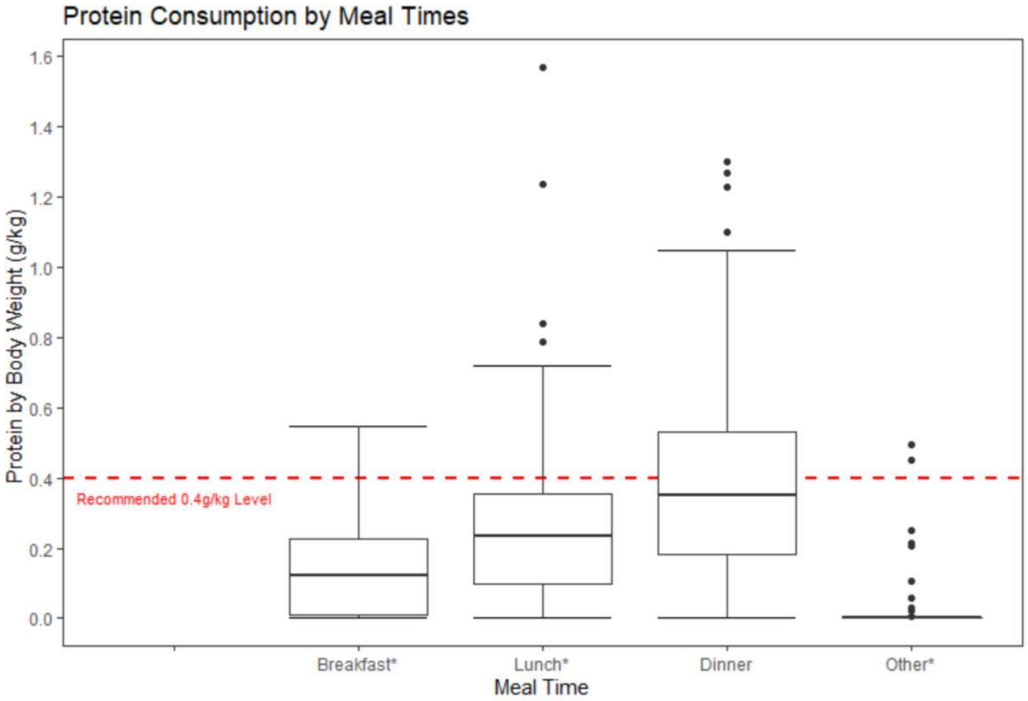

Figure S1. Protein consumption by meal times (g protein/kg BW). Per meal consumption of protein per kg of body weight was: breakfast 0.14 ± 0.1, lunch 0.28 ± 0.3, dinner 0.39 ± 0.3, and other 0.02 ± 0.1. Protein consumption at breakfast, lunch, and other were significantly different (p<0.0001) than the 0.4g/kg threshold, while dinner was not significantly different (p=.736). Only 3.3% of participants met the threshold at breakfast, 22.8% at lunch, and 42.4% at dinner. One participant (1.1%) met the 0.4 g/kg threshold for three meals, 13 (14.3%) met the threshold for two meals, 51 (55.4%) met it for at least one meal, and 41(44.6%) of the participants did not meet the threshold at any meal.

Table S1. Protein consumption meeting per meal recommendation (g/kg BW) by meal time.

**Meal**	**Met per meal recommendation ≥0.4g/kg; n(%)**
Breakfast	3 (3.3%)
Lunch	21 (22.8%)
Dinner	39 (42.4%)
Other	2 (2.2%)

Notes: g, grams kg, kilograms.

Table S2. Average protein consumption to weight comparison of current recommendations (g/kg).

**Recommendations (Protein g/kg BW)**	**Average consumption**	**Range**	**Met recommendations, n(%)**	**p-value**
Current RDA (0.8 g/kg) [13]	0.83 ± 0.4	0.10 – 2.78	39 (43.4%)	0.460
Proposed standard (1.2 g/kg)[16]	0.83 ± 0.4	0.10 – 2.78	16 (17.4%)	**<0.0001**

Notes: p-value <0.05 indicates statistical significance across groups. RDA, recommended daily allowance; BW, body weight; kg, kilograms.
